# SARS-CoV-2/COVID-19-Auswirkungen auf die Plazenta

**DOI:** 10.1007/s00292-021-00952-7

**Published:** 2021-06-11

**Authors:** T. Menter, A. Tzankov, E. Bruder

**Affiliations:** Pathologie, Institut für Medizinische Genetik und Pathologie, Universitätsspital Basel, Universität Basel, Schönbeinstrasse 40, 4031 Basel, Schweiz

**Keywords:** Angiotensin-konvertierendes Enzym 2, Coronavirusinfektion, Plazenta, Schwangerschaft, Vaskulitis, Angiotensin-converting enzyme 2, Coronavirus infection, Placenta, Pregnancy, Vasculitis

## Abstract

Ein besonderes Augenmerk bei der durch das Severe-acute-respiratory-syndrome-Coronavirus‑2 (SARS-CoV-2) hervorgerufenen Coronaviruskrankheit 2019 (COVID-19) wurde von Beginn an auf die Gruppe der Schwangeren gelegt.

Nach einer Einführung zur Immunabwehr der Plazenta und viralen plazentaren Infektionen, beschreiben wir die morphologischen Veränderungen der Plazenta bei SARS-CoV-2-Infektion der Mutter, ziehen Vergleiche zur SARS-Epidemie und diskutieren die Frage der vertikalen Transmission von SARS-CoV‑2 von der Mutter auf das Neugeborene.

Die häufigsten pathologischen Befunde der Plazenta bestehen in Zeichen der maternalen und auch fetalen Malperfusion sowie immunologisch bzw. thromboinflammatorisch vermittelten Veränderungen. Es finden sich Infarkte, deziduale Vaskulopathie sowie Thromben im fetalen Kreislauf und Vermehrung avaskulärer Villi. Daneben zeigen sich in einigen Fällen Entzündungsreaktionen mit Villitis und Intervillositis sowie eine Vaskulitis fetaler Gefäße. Zudem konnte der Nachweis erbracht werden, dass SARS-CoV‑2 die Plazenta direkt infizieren kann. Somit ist auch eine vertikale Transmission möglich.

Ein COVID-19-spezifisches Schädigungsmuster der Plazenta liegt bislang nicht vor, obwohl der Nachweis von fetaler Thrombovaskulitis, Villitis und Intervillositis sowie einer fetalen und maternalen Malperfusion in Analogie zu der bereits bekannten allgemeinen Pathophysiologie von COVID-19 (Entzündungsreaktion und Mikrozirkulationsstörung) interpretiert werden könnte. Der Nachweis viraler RNA in den fetalen Kompartimenten der Plazenta/der Nabelschnur zeugt von der vertikalen SARS-CoV‑2 Transmission.

## Abstract (en)

From the very beginning, special attention regarding severe acute respiratory syndrome-coronavirus‑2 (SARS-CoV-2) and the resulting coronavirus disease-2019 (COVID-19) has been paid to pregnant women.

In this review, after a short introduction into the immunodefensive role of the placenta and viral infections in general, we describe the morphological changes of the placenta in SARS-CoV-2-infected pregnant women based on our own and other published studies, draw comparisons to the SARS epidemic, and discuss the question of vertical transmission of SARS-CoV‑2 from the mother to the neonate.

The most common pathological findings of the placenta in SARS-CoV‑2 infection are signs of maternal and fetal malperfusion as well as potentially immunologically and/or thromboinflammation-mediated findings. These manifest as infarcts and decidual vasculopathy as well as thrombi in the fetal circulation and avascular villi. In some cases, there is also an inflammatory reaction with villitis, intervillositis, and fetal vasculitis. In addition, it has been shown that SARS-CoV‑2 can directly infect the placenta, so vertical transmission is possible.

There is no COVID-19 specific pattern of placental alterations, although the detection of fetal thrombovasculitis, villitis, and intervillositis as well as fetal and maternal malperfusion could be best interpreted as the signature of SARS-CoV‑2 infection – considering the known pathophysiology of COVID-19 regarding other organs (inflammatory reaction and [micro]angiopathy). Detection of viral RNA in the fetal placental tissue and the umbilical cord indicates SARS-CoV‑2 vertical transmission.

Schon früh wurde im Rahmen von Severe-acute-respiratory-syndrome-Coronavirus-2(SARS-CoV-2)-Infektionen und der dadurch bedingten Coronaviruskrankheit 2019 (COVID-19) diskutiert, ob Schwangere eine Risikogruppe darstellen und ob darüber hinaus die Gesundheit und Entwicklung des ungeborenen Kindes bedroht sein kann [1, 2]. Auch schon bei der Spanischen Grippe 1918/19 stellten Schwangere eine explizite Risikogruppe dar [3].

Zahlen größerer Studien zeigen, dass die SARS-CoV-2-Infektionsrate bei Schwangeren höher liegt als in der Allgemeinbevölkerung [4]. Zudem stellt eine Zusammenfassung mehrerer publizierter Studien eine erhöhte Frühgeburtsrate von 37 % und ein perinatales Versterben bei 2 von 67 Kindern fest [5]. Pneumonien bzw. Atemnotprobleme wurden bei 18 % der Kinder beschrieben. Gemäß einer großen spanischen Studie verläuft erfreulicherweise COVID-19 bei Neugeborenen meist milde [6].

Nach einer kurzen Einführung zum Thema über die allgemeine Immunreaktion der Plazenta und ihrer Reaktion bei anderen viralen Infektionen, fassen wir die bislang beschriebenen morphologischen Veränderungen der Plazenta im Rahmen mütterlicher SARS-CoV-2-Infektion zusammen und diskutieren deren Auswirkungen auf die Schwangerschaft und die Gesundheit von Mutter und Kind. Zudem diskutieren wir die Frage der vertikalen Transmission von SARS-CoV‑2.

## Immunabwehr und Infektionen der Plazenta

Die Funktion als Barriere und Immuntoleranzvermittler zwischen mütterlichem und kindlichem Organismus ist, neben der Ernährung des Kindes, eine der wichtigsten Aufgaben der Plazenta, welche diesbezüglich als sog. immunprivilegiertes Organ anzusehen ist. Die Mechanismen der Immunabwehr der Plazenta sind vielfältig: An erster Stelle steht hierbei die Dezidua. Der Synzytiotrophoblast, der die Zottenoberfläche bekleidet, stellt eine starke physische Barriere für Pathogene dar [7–9]. Auch typische Oberflächenmoleküle wie E‑Cadherin, die Viren als Zelleintrittspforte dienen können, werden hier nur gering bis nicht exprimiert [10]. Toll-like-Rezeptoren, die eine wichtige Rolle im Rahmen der angeborenen Immunität („innate immunitiy“) spielen, aber auch tolerogene Moleküle wie PDL1, werden von der Plazenta, je nach Schwangerschaftsmonat, verschieden exprimiert und tragen zur Balance zwischen Toleranz des kindlichen Gewebes durch das mütterliche Immunsystem und eine adäquate Virusabwehr bei [11]. Der Synzytiotrophoblast kann verschiedene microRNAs, darunter das Chromosom-19-miR-Cluster (C19MC) sezernieren, was die Autophagie und damit die Elimination virusinfizierter Zellen stimuliert [12]. Eine weitere wichtige Rolle kommt der Typ-III-Interferonantwort zu, deren protektiver Wert auch schon bei anderen Coronaviren (Severe-acute-respiratory-syndrome[SARS]-Coronavirus und Middle-east-respiratory-syndrome[MERS]-Coronavirus) beschrieben wurde [13]. Der dritte wichtige Mechanismus ist der NF-κB Signalweg im Trophoblast und vor allem dessen proinflammatorische Eigenschaften [14].

Trotz dieser Abwehrmechanismen kommt es bei vielen Viren zu einer vertikalen Übertragung von der Mutter auf das Kind [15]. Dazu trägt bei, dass im ersten Trimenon die oben erwähnten Mechanismen noch nicht vollständig ausgebildet sind, während am Ende der Schwangerschaft bereits eine Degeneration des Synzytiotrophoblast beginnt [16]. Darüber hinaus können auch eine Immundefizienz der Mutter oder gestationsassoziierte Erkrankungen (z. B. Eklampsie/Gestose) die Abwehrfunktion der Plazenta reduzieren [17] und zu einer Störung der Abwehrfunktion der Plazenta beitragen. Die klassischen Erreger sind in der TORCH-Gruppe zusammengefasst: *Toxoplasma gondii*, andere („others“) (darunter Varicella-Zoster-Virus, Parvovirus B19, humanes Immunodefizienzvirus [HIV], Enteroviren, *Listeria monocytogenes, Treponema pallidum*), Rötelnvirus, Zytomegalievirus (CMV) und Herpes-simplex-Virus (HSV). Seit kurzer Zeit zählt auch das Zikavirus hierzu [18]. Teilweise zeigen durch diese Erreger befallene Plazenten eine typische Morphologie: z. B. lymphoplasmazelluläre Villitis und Intervillositis bei CMV- und HSV-Infektionen [19] oder Proliferation der Hofbauer-Zellen (spezialisierte plazentare Makrophagen) bei der Zikai-Infektion [20]. Um die natürlichen Barrieren der Plazenta zu umgehen, nutzen verschiedene Viren unterschiedliche Mechanismen. So bedient sich CMV der Transzytose, mittels der ansonsten Immunglobuline durch den Synzytiotrophoblasten geschleust werden [21], während HIV und das Zika-Virus Makrophagen befallen und sich dort replizieren [10].

## Erfahrungen aus den SARS- und MERS-Epidemien

Auch bei den zurückliegenden epidemischen Ausbreitungen der Coronavirus-assoziierten Erkrankungen SARS und MERS wurden schwangere Frauen als potenzielle Risikogruppen eingeordnet. Bei SARS konnte gezeigt werden, dass die Rate intensivpflichtiger Behandlungen der Mütter und die mütterliche Sterberate deutlich erhöht waren, zudem kam es zu einer erhöhten Rate von Fehl- und Frühgeburten sowie intrauterinen Fruchttoden [22]. Bei MERS konnte – bei insgesamt wenigen in der Literatur beschriebenen Fallserien – eine deutlich erhöhte maternale Mortalität von 40 % nachgewiesen werden, zudem war auch die Rate der Frühgeburtlichkeit und auch der Präeklampsie erhöht [23]. Eine vertikale Transmission von SARS und MERS konnte bislang nicht belegt werden [24].

Die Plazentahistologie ist bisher nur im Rahmen einer Studie von 7 SARS-Patientinnen aus Hongkong veröffentlicht [25]. Die Hauptbefunde waren eine maternale und fetale Malperfusion, Entzündungsinfiltrate wurden nicht festgestellt. Beschreibungen zu Plazenten von MERS-Patientinnen liegen bisher nicht vor. In der Literatur findet sich ein Fallbericht über eine vorzeitige Plazentalösung ohne Informationen bezüglich einer histologischen Aufarbeitung [26].

## Morphologische Plazentaveränderungen bei SARS-CoV-2-positiven Schwangeren

Bei der Beschreibung morphologischer Veränderungen von Plazenten bei SARS-CoV-2-positiven Schwangeren müssen verschiedene Aspekte beachtet werden. Neben der Frage, ob eine Patientin lediglich den positiven Nachweis von SARS-CoV‑2 im Nasopharyngealabstrich hat oder ob sie an einer manifesten COVID-19-Erkrankung leidet, ist auch die Frage zu klären, ob die Erkrankung zum Zeitpunkt der Geburt und damit in aktuellem Bezug zur morphologischen Beurteilung der Plazenta oder weiter in der Schwangerschaft zurückliegt. Bei letzterer Konstellation kann die Assoziation zwischen SARS-CoV-2/COVID-19 und der Morphologie schwieriger zu interpretieren sein, da die Veränderungen der Plazenta multifaktoriell sein können. In dieser Übersichtsarbeit fokussieren wir uns primär auf Veränderungen, die bei einer akuten Infektion mit SARS-CoV‑2 zum Zeitpunkt der Geburt beschrieben sind.

Zahlreiche Studien konnten zeigen, dass bei SARS-CoV-2-Infektionen neben entzündlich-immunologisch vermittelten Reaktionen auch das Risiko mikrozirkulatorischer Störungen besteht [27, 28]. Passend hierzu, lassen sich die an der Plazenta in den bisher publizierten Studien erhobenen Befunde in 2 Gruppen einteilen: Entzündungsinfiltrate in Form einer Villitis/Perivillitis oder auch Intervillositis sowie Zeichen einer maternalen und/oder fetalen Malperfusion [4].

Mehrfach konnte SARS-CoV‑2 in den Plazentazotten nachgewiesen werden [29–31]. Der Goldstandard ist hierbei der Nachweis mittels PCR. Auch immunhistochemisch lassen sich z. B. das S‑ oder N‑Antigen von SARS-CoV‑2 nachweisen, hierbei ist jedoch auf entsprechende Färbeartefakte zu achten und eine adäquate Austestung im eigenen Labor unabdingbar [32]. Diese Studien und mehrere Übersichtsarbeiten [4, 33] berichten auch über eine Villitis bzw. Intervillositis, sodass davon ausgegangen werden kann, dass die Entzündung virusassoziiert bzw. -verursacht sein könnte. Hierfür wurde der Terminus „SARS-CoV-2-Plazentitis“ geprägt [34]. In einem besonderen Fall aus einer Fallserie konnte gezeigt werden, dass bei einer solchen SARS-CoV-2-Plazentitis dasselbe Genexpressionsprofil wie im COVID-19-Lungenparenchym vorliegt, was auf die Bedeutung der COVID-19-typischen thromboinflammatorischen Reaktion mit potenzieller schädlicher Auswirkung auf das Kind auch ohne vertikale Transmission hinweist (in diesem Fall zeigte sich, die Hypothese stützend, eine perinatale Asphyxie mit neurologischem Defizit des Kindes) [35]. In weiteren Studien wurden keine spezifischen Assoziationen einer chronischen Villitis bei manifester COVID-19-Erkrankung gefunden. Eine weitere Differenzialdiagnose bleibt auch die Villitis unklarer Ätiologie (VUE), welche auf immunologische Phänomene („graft-versus-host-like“) verweist [36]. Andere Studien hingegen halten eine chronische Villitis in Patientinnen ohne manifeste COVID-19-Erkrankung fest und berichten, im Vergleich zu adäquaten Kontrollkohorten, über keine gesteigerte Inzidenz der Villitis/Intervillositis [36–39]. Natürlich bleibt die chronische Villitis unklarer Ätiologie („villitis of unknown origin“) immer eine Differenzialdiagnose, jedoch scheint eine Assoziation „Entzündung & SARS-CoV-2“ gerade auch im Hinblick auf die Morphologie anderer Virusinfektionen gut möglich. Die selten zu diagnostizierende Intervillositis ist in den meisten Fällen mit Autoimmunität assoziiert, in Einzelfällen wird eine Assoziation mit Erregern diskutiert [40]. Die entsprechenden Fälle berichten nicht über eine vorhandene Autoimmunkrankheit der Mutter, sodass die Assoziation der Intervillositis – insbesondere bei entsprechendem Nachweis von SARS-CoV‑2 im Plazentagewebe – mit einer SARS-CoV-2-Infektion plausibel ist, vor allem, da bei COVID-19 eine Induktion eines Autoimmunphänomens analog dem Antiphospholipidsyndrom nachgewiesen werden konnte [41].

**Figure 1:**
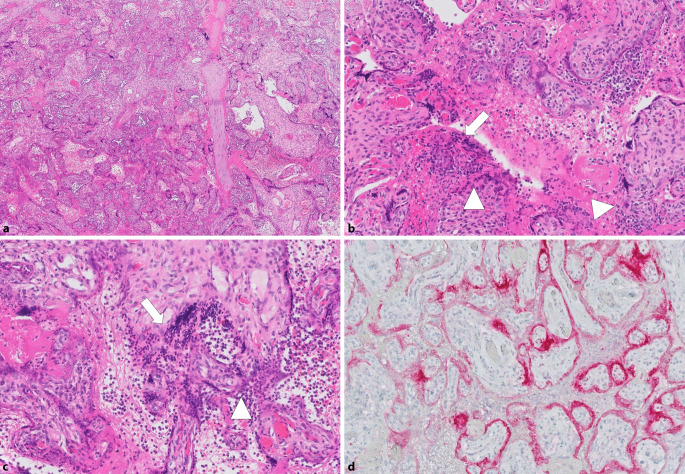

**Figure 2:**
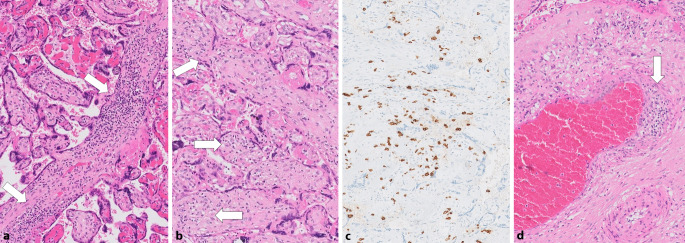

In unserem Untersuchungsgut der letzten 13 Monate fanden wir 2 Fälle mit der oben beschriebenen „Plazentitis“ im Sinne einer Intervillositis mit prominenten Nekrosen des Synzytiotrophoblasten und Nachweis von SARS-CoV‑2 mittels PCR und Immunhistochemie (siehe Abb. 1). Ein weiterer Fall aus der ersten COVID-19-Welle war eine symptomatische Patientin mit Virusnachweis in der PCR und der in-situ-Hybridisierung und deutlich ausgeprägter, chronischer CD8-positiver lymphozytärer Villitis (Abb. 2a–c) sowie einer Vaskulitis der Stammzottengefäße mit Thrombenbildung in der Plazenta (Abb. 2d; [42]). Bei zahlreichen anderen asymptomatischen Patientinnen mit lediglich SARS-CoV‑2 positivem Nasopharyngealabstrich ließen sich diese Befunde nicht nachweisen. Die von uns beschriebene Vaskulitis fetaler Gefäße in größeren Stammzotten wurde in anderen Studien bislang nicht beschrieben.

Dennoch ist ein weiterer wichtiger, wiederkehrender Befund eine maternale und teils auch fetale Perfusionsstörung [37, 38, 43]. Diese kann zu einer Beeinträchtigung der plazentaren Versorgungsleistung und somit zu einer Gefährdung des ungeborenen Kindes führen und wird analog des internationalen Konsensus (Amsterdam-Klassifikation) diagnostiziert [44]. Zeichen der maternalen Perfusionsstörung sind, neben Infarkten, auch eine Vorreifung des Plazentaparenchyms sowie die deziduale Vaskulopathie, Tenney-Parker-Veränderungen (Vermehrung synzytialer Kernknospen der Villi) und retroplazentare Hämatome. Während vor allem die massive perivillöse Fibrinablagerung in erster Linie Ausdruck einer pathologischen Immunreaktion ist [45], kann eine Vermehrung des perivillösen Fibrins auch im Rahmen der fetalen Malperfusion interpretiert werden [46]. Als Zeichen fetaler Malperfusion werden Thromben im fetalen Kreislauf sowie avaskuläre Villi, Zeichen der Karyorrhexis (betroffen sind villöse Stromazellen und/oder Endothelien und kernhaltige Erythrozyten sowie Leukozyten) klassifiziert. Die Chorangiose ist primär Folge einer niedriggradigen Hypoxie [47] und korreliert mit einer erhöhten kindlichen Morbidität und Mortalität und auch einer fetalen Malperfusion [48]. Hier ist es wichtig, den Einfluss von SARS-CoV‑2 von anderen klassischen Ursachen maternaler und fetaler Malperfusion wie schon vorbestehender maternaler Hypertonie und dem in der Schwangerschaft *per se* alterierten Gerinnungsstatus zu unterscheiden [49]. Eine Fallserie berichtet von einer gesteigerten Inzidenz fetaler Malperfusion, gekennzeichnet durch Thrombosen und avaskuläre Villi [43]. Diese Befunde wurden als Beleg für die systemischen prokoagulatorischen Veränderungen im Rahmen der SARS-CoV-2-Infektion gewertet, passend zu Veränderungen in anderen Organen [28].

Einzelne der angeführten Fallberichte beschreiben Komplikationen der Neugeborenen oder sogar Totgeburten bei ausgeprägter Intervillositis/Villitis oder schwerer Malperfusion der Plazenta auch ohne Nachweis der im nächsten Abschnitt diskutierten vertikalen Transmission [31, 35, 50]. Bei den von uns untersuchten Fällen waren die Neugeborenen klinisch unauffällig und auch negativ für SARS-CoV‑2. In mehreren bisher vorliegenden Metaanalysen bzgl. der Neugeborenen SARS-CoV-2-positiver Mütter fanden sich keine ausreichenden Angaben zur Plazentamorphologie [51, 52]. Somit scheint es zur abschließenden Korrelation zwischen Plazentamorphologie und etwaigen Befunden der Kinder noch zu früh.

## Vertikale Transmission und Mechanismen der SARS-CoV-2-Infektion der Plazenta

Mehrere sorgfältig aufgearbeitete Einzelbeschreibungen einer vertikalen Transmission sind bisher belegt [53]. Hierbei konnte SARS-CoV‑2 mittels verschiedener Untersuchungen des Plazentagewebes, darunter der Nabelschnur, auch im kindlichen Gewebe nachgewiesen werden. Die vertikale Transmission wird durch eine massive Virämie der Mutter und/oder durch eine entsprechende Vorerkrankung, wie z. B. eine Präeklampsie als primär immundeprivierender Faktor, begünstigt [29].

**Figure 3:**
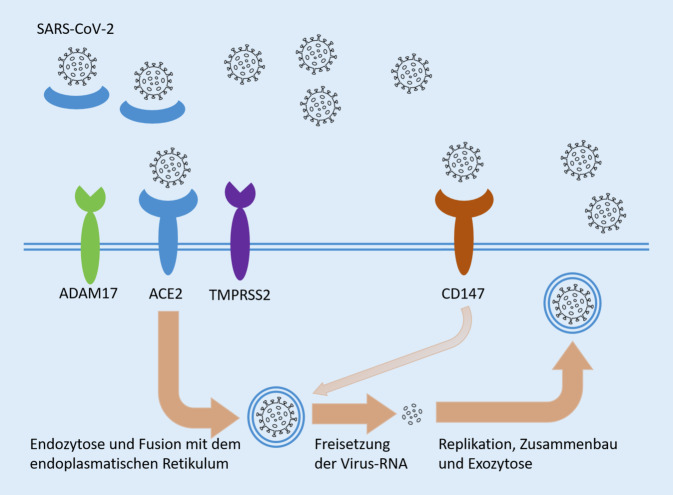

Als Haupteintrittspforte für SARS-CoV‑2 in Zellen gilt aktuell das Angiotensin-konvertierende Enzym 2 (ACE2). Zudem ist das Vorhandensein weiterer Oberflächenproteine und intrazellulärer Proteine, wie z. B. humane Transmembranprotease Serin 2 (TMPRSS2) oder Cathepsin L und Blutgruppenantigen A, für die Spaltung des S‑Proteins notwendig [54]. Daneben werden auch alternative Zellinvasionsmechanismen von SARS-CoV‑2 diskutiert, darunter CD147 [55]. Einen Überblick über die Zellinvasionsmechanismen und die Replikation von SARS-CoV‑2 gibt Abb. 3. Darüber, wie und wann diese Proteine in Zellen der Plazenta exprimiert werden, herrscht noch eine gewisse Unklarheit. Die Expression von ACE2 wird seit vielen Jahren in der Plazenta erforscht, da das Renin-Angiotensin System, zu dem auch ACE2 gehört, eine wichtige Rolle bei der Angiogenese und der Trophoblastinvasion spielt [56, 57]. In den von uns untersuchten Fällen und einer adäquaten Kontrollgruppe sahen wir jedoch lediglich eine schwache Expression von ACE2 in dezidualen Zellen und dezidualisiertem Endometrium, allerdings nicht im Synzytiotrophoblast oder anderen fetalen Zellen. CD147 hingegen scheint in der Plazenta konstant und in vielen Zelltypen exprimiert zu sein [58] und könnte somit einen alternativen SARS-CoV-2-Andockmechanismus darstellen [59]. Eine weitere aktuelle Studie berichtet im Gegensatz zu den bisher vorliegenden Ergebnissen in allen untersuchten Fällen einen Nachweis von SARS-CoV-2-Spikeprotein im Synzytiotrophoblast in Kombination mit einer hohen ACE2-Expression. Andere Methoden des Virusnachweis wurden in dieser Studie jedoch nicht erbracht [60] und der verwendete Antikörperklon wird kontrovers diskutiert [61].

## Fazit für die Praxis

- Ein Durchdringen der plazentaren Barriere und Infektion des ungeborenen Kindes durch SARS-CoV‑2 ist möglich, aber nach gegenwärtigem Kenntnisstand selten.
- Die Interpretation morphologischer Befunde bei SARS-CoV-2-Infektion kann mittels Zusatzuntersuchungen (PCR, Immunhistochemie, In-situ-Hybridisierung) verbessert werden.
- Die fetale Thrombovaskulitis, Villitis und Intervillositis sowie die fetale und maternale Malperfusion können analog zu der bereits bekannten Pathophysiologie von COVID-19 (Entzündungsreaktion [SARS-CoV-2-Plazentitis] und Mikrozirkulationsstörung) interpretiert werden. Ein COVID-19-spezifisches Schädigungsmuster der Plazenta liegt bislang jedoch nicht vor.
- Weitere Studien an größeren Kollektiven und mit klar definierten morphologischen, serologischen und klinischen Parametern sind notwendig, um die Affektion der Plazenta durch SARS-CoV-2/COVID-19 besser verstehen und einschätzen zu können.
